# “Sex Should Not Be Part of the Lives of Persons with Disabilities, but They Are Human Beings Too”: Perceptions of Healthcare Providers and Factors Affecting Service Delivery in Ghana

**DOI:** 10.3390/healthcare11071041

**Published:** 2023-04-04

**Authors:** Abdul-Aziz Seidu, Bunmi S. Malau-Aduli, Kristin McBain-Rigg, Aduli E. O. Malau-Aduli, Theophilus I. Emeto

**Affiliations:** 1 Public Health & Tropical Medicine, College of Public Health, Medical and Veterinary Sciences, James Cook University, Townsville, QLD 4811, Australia; 2 Department of Population and Health, University of Cape Coast, Cape Coast P.O. Box UC 182, Ghana; 3 College of Medicine and Dentistry, James Cook University, Townsville, QLD 4811, Australia; 4 School of Medicine and Public Health, The University of Newcastle, Newcastle, NSW 2308, Australia; 5 School of Environmental and Life Sciences, The University of Newcastle, Newcastle, NSW 2308, Australia; 6 World Health Organization Collaborating Center for Vector-Borne and Neglected Tropical Diseases, James Cook University, Townsville, QLD 4811, Australia

**Keywords:** attitude, Ashanti region, Ghana, persons with disabilities, sexual and reproductive healthcare

## Abstract

Persons with disabilities (PwDs) constitute about 16% of the global population and face many challenges in every society, including access to sexual and reproductive healthcare. The attitudes of healthcare providers (HPs) exert a major influence on PwDs accessing sexual and reproductive healthcare (SRH). A sequential explanatory mixed methods design was used to investigate the attitudes and perceptions of HPs towards PwDs and SRH delivery in Ghana’s Ashanti region. Quantitative data analysis indicated that overall, 82% of HPs had received information on disability-related issues and had relatively positive attitude towards PwDs, which varied across sub-scales of the Attitude Towards Disability score and associated with their sociodemographic characteristics. HPs faced several challenges in SRH services delivery to PwDs, which included a lack of funding and training, and inadequate staff. Inductive thematic analysis of the qualitative data revealed eight overarching themes. The findings revealed that HPs had prejudice about the mental and sexual health abilities of PwDs. Inadequate skill set, inadequate resources, and limited funding were major challenges identified. Nonetheless, compassion and benevolence towards PwDs, improvision, economic and educational support, awareness creation, and referrals were strategies adopted to overcome these challenges. Mandatory training of HPs is recommended to ensure improved SRH service delivery to PwDs. Future research could explore the perceptions and coping strategies of PwDs.

## 1. Introduction

Persons with disabilities (PwDs) constitute about 16% of the world’s population [1]. However, they experience significant difficulties in various aspects of their lives, including access to sexual and reproductive health (SRH) services [1,2]. Consequently, healthcare providers (HPs) are vital to the provision of essential and improved SRH services to PwDs. In high-income countries (HICs), various interventions are implemented to ensure that HPs are trained to provide vital services, including SRH services to PwDs [3]. In contrast, the challenges PwDs face in low-and middle-income countries (LMICs) are exacerbated by a combination of physical, financial, and attitudinal factors [1].

Evidence suggests that the attitudes of HPs towards PwDs have significant effects on the type and nature of the care they provide to PwDs. Attitude is defined as “the combination of beliefs and feelings held by an individual that predisposes them to behave in a certain way towards another person. It comprises affective, cognitive, and behavioural components” ([4], p. 2). The attitude of HPs toward PwDs may directly and/or indirectly discourage PwDs from accessing SRH services, thus contributing to the already existing plethora of other barriers faced in SRH accessibility [2,5,6,7]. Two studies reported the positive attitudes of 56.5% [8] and 50% [9] of HPs towards PwDs; in contrast, the study by Devkota et al. [4] showed that HPs’ attitudes towards PwDs were negative, accompanied by a lack of knowledge and capabilities in service provision. Others have reported that HPs’ personal characteristics influenced their attitudes toward PwDs; however, the results were inconclusive [10,11,12]. For example, women were found to have higher positive attitudes than men [10,11,12], while the reverse was found in other studies [4,13]. Similar conflicting results have been reported with respect to the place of residence, age, level of exposure, and contact with PwDs [4,14,15].

Some studies have discussed the probable reasons for the negative attitude of HPs towards PwDs. The predominant reasons include misconceptions, inadequate knowledge and training, and discomfort when working with PwDs [4,5]. For instance, women with disabilities may not be asked about contraception, and HPs may put off a pelvic exam because they believe women with disabilities are often not sexually active [16,17]. Persons with visual impairments have also been reported to be ridiculed by HPs because they requested HIV/AIDs testing services [18].

Disability care and management are rarely discussed in health training schools, especially in LMICs [4]. Some medical schools in HICs include disability issues in their curriculum to improve students’ knowledge, attitude, and competencies in disability care, but it is still less prioritised [19]. The situation is even worse in LMICs. In Zambia, for instance, inadequate knowledge about disability care was identified among the factors that prevented HPs from providing adequate care to children with disabilities [20]. In Ethiopia, a lack of awareness, education, and training among HPs has resulted in persons with intellectual disabilities being undiagnosed [21].

In LMICs, HPs are critical in providing information and preventive, clinical, and rehabilitative services to the populace [4]. Yet, HPs have had little formal training in disability care and management despite the provisions of the United Nations Convention on the Rights of Persons with Disabilities (UNCRPwD). In Ghana, the 2006 Disability Act 715 mandates HPs to receive adequate training on the care for PwDs. Although sign language has been introduced in some health training institutions, Acheampong et al. [16] reported its ineffectiveness. Moreover, there is still a low level of inclusion of disability-related issues in SRH health policies [22,23]; evidence suggests that PwDs are relatively better cared for in HICs than in LMICs [1,24]. To improve the delivery of SRH services to PwDs in LMICs, it is important to explore the perceptions of HPs in both rural and urban areas. Therefore, this study focused on Ghana’s Ashanti Region and sought to answer the following research questions: (a) What factors influence the attitude of HPs towards PwDs? and (b) What are the enablers and barriers to HPs delivery of SRH services to PwDs?

### 1.1. Theoretical Framework 

**Figure 1:**
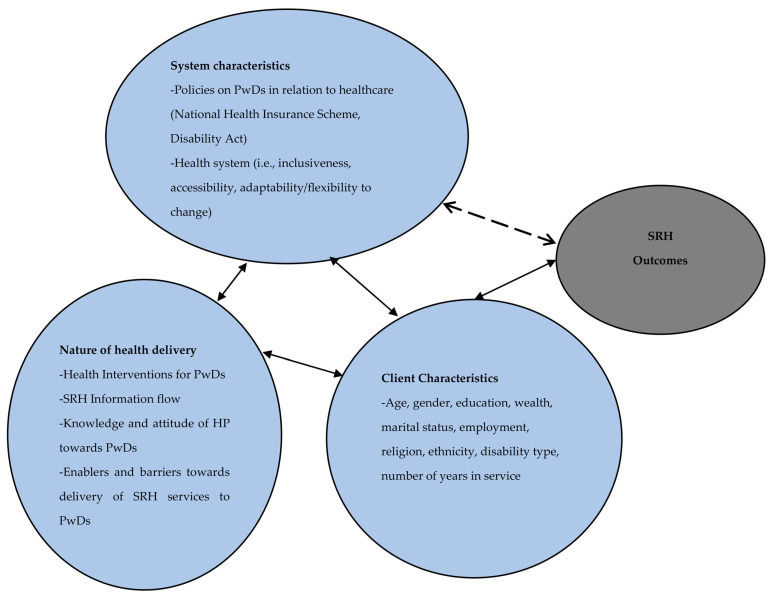
Health outcomes model. Source: Adapted from Mitchell et al. [25] and Radwin [26].

This study is situated within a broader mixed methods project entitled “Impact of health policies and interventions on the sexual and reproductive health outcomes among persons with disabilities in Ghana”. The broader study is guided by the Quality Health Outcomes Model adapted from Mitchell [25] and Radwin [26]. Figure 1 explains how the components of the model–system characteristics, interventions, and client characteristics influence health outcomes or behaviours. The model depicts that there is an interconnection of both system and individual characteristics in producing desirable health outcomes. This signifies that no single policy/intervention acts directly through either the system or client alone. The effect/outcome of a policy/intervention is influenced by the characteristics of the client and the health system [27]. 

#### 1.1.1. System Characteristics 

The system characteristics portray how the health system is perceived as an organised agency, whether as a hospital or HP network, and how these traits interact to influence an individual’s health and behaviour. In this study, the system characteristics comprise the nature of the health system, its inclusiveness, accessibility, adaptability/flexibility to change, health administration, and the policies on PwDs in relation to SRH healthcare access. The nature of health delivery, such as the specific things HPs do for PwDs when they visit a health facility, the SRH information flow and access, knowledge and attitude of HPs towards PwDs, and how all these attributes serve as enablers or disabling factors towards the delivery of SRH services to PwDs [28]. 

#### 1.1.2. Interventions

In the model, the interventions include direct and indirect measures and related activities by government, HPs, and non-governmental organisation (NGO) officials to improve the SRH of PwDs. The policies or interventions include the national health insurance scheme, free maternal healthcare policy, Disability Act 715, Livelihood Empowerment Against Poverty, and Disability Common Fund. 

#### 1.1.3. Client Characteristics

The client characteristics are the factors related to the individual, including the person’s demographic characteristics. In this study, client characteristics include the sociodemographics of HPs and PwDs, such as age, marital status, number of years in the service, professional qualification, type of health facility, and location of the health facility. With the HPs, these characteristics have an influence on their attitude toward PwDs in SRH services delivery. 

#### 1.1.4. Outcomes

The model outcomes include both positive and adverse SRH conditions that an individual can experience [27]. In the current study, these include the PwDs’ self-reported SRH problems such as sexually transmitted infections, unplanned pregnancy, self-rated SRH, sexual safety, sexual autonomy, sexual satisfaction, and experience of gender-based violence. The attitude of HPs towards PwDs is a key determinant of policy implementation and interventions aimed at improving the SRH outcomes of PwDs. The current study focused on three aspects of the framework—system characteristics, client characteristics, and the nature of health delivery (Figure 1). The client characteristics aspect of the framework has an influence on the attitude of HPs, and the nature of the health delivery system also influences their attitudes toward PwDs. Use of the service is determined by the interactions between the characteristics of the population and that of the healthcare system [29]. Thus, access to SRH is achieved if health resources fit or interact with the individual’s healthcare needs and are delivered appropriately by HPs. It is an ideal model for this study because it has been validated [26] and has been used in several settings in different populations [30]. It is also a framework for analysing health outcomes in relation to systems, interventions, and individual characteristics [30], and all these constructs are the key issues to be assessed within the broader study.

## 2. Materials and Methods

### 2.1. Ethics

Before the commencement of the study, ethical approvals from the Ghana Health Service (GHS) Ethics Review Board (GHS-ERC:005-0621), Komfo Anokye Teaching Hospital (KATH-IRB/RR/101/21), and James Cook University Human Research Ethics Committee (JCUHREC-H8531) were obtained. Further endorsement of the ethics approval documents was obtained from Offinso North District Health Directorate, Akumadan, and all the health facilities. Before data collection commenced, respondents gave both verbal and written informed consent. All procedures were in accordance with the Helsinki Declaration on ethical procedures in conducting research among human subjects.

### 2.2. Study Setting and Target Population

**Figure 2:**
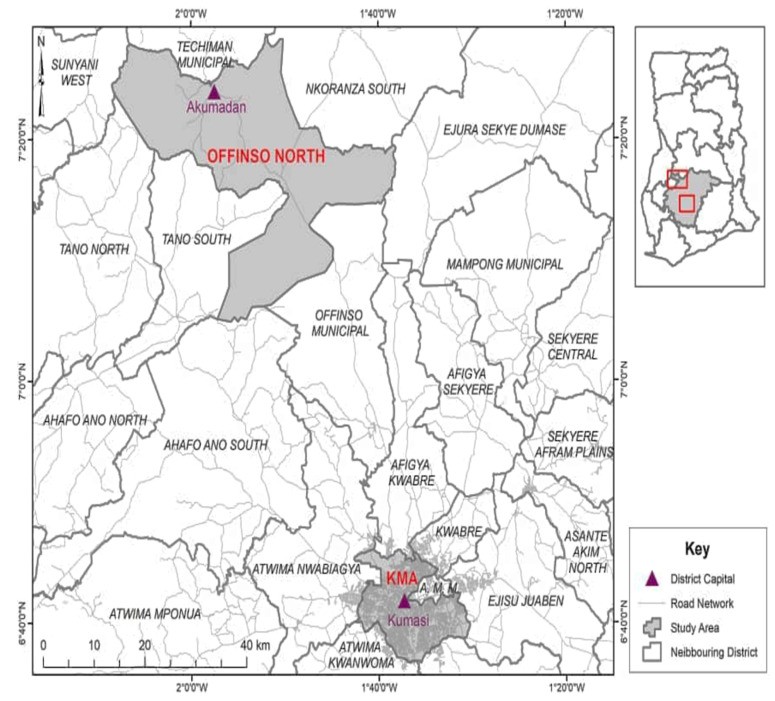
Map of the study area. Source: GIS [32].

The study was conducted in the Ashanti Region, Ghana’s most populated region, with the highest percentage (17.3%) of PwDs [31] and located in the central belt between longitudes 0.15° W and 2.25° W and latitudes 5.5° N and 7.46° N. It shares borders with Bono East, Central, Eastern, and Western Regions to the north, south, east, and west, respectively (Figure 2). It covers about 24,389 square kilometres, equivalent to 10.2% of Ghana’s land area. About 61.6% of the Ashanti region is urbanised. It is organised administratively into 43 Metropolitan, Municipal, and District Assemblies. There is 1 metropolis, 18 municipalities, and 24 districts [31]. The region was chosen for this study due to its population size, strategic location, and cultural diversity. To incorporate both rural and urban perspectives in the study, one rural (Offinso North District) and one urban area (Kumasi metropolis) were chosen for the study.

The study involved HPs (doctors, nurses, and midwives) because they are the major SRH service providers. The inclusion criteria were: (a) HPs who had worked for 12 months and above, and (b) the health worker being a nurse, doctor, or midwife. Data were collected from 11 health facilities comprising 6 major government-owned and 2 NGO-owned facilities in the Kumasi metropolis, and 3 in the Offinso North District.

### 2.3. Study Design

The study is grounded in the sequential explanatory mixed method study design [33]. The first phase of the study involved quantitative data collection and analysis. This was subsequently followed by qualitative data collection and analysis to offer detailed explanations of some of the issues that emerged in the quantitative phase. For the quantitative phase, a cross-sectional study was conducted among HPs. The findings of the quantitative phase informed the development of the interview guide for the qualitative phase of the study. A phenomenological approach was employed in the qualitative phase for HPs to share their experiences, beliefs, perceptions, and attitudes on providing SRH care to PwDs. Consolidated criteria for reporting qualitative research (COREQ) were also followed in reporting the qualitative aspect of the research [34].

### 2.4. Data Collection

The quantitative data collection period was from December 2021 to March 2022, while the qualitative phase lasted from April to June 2022. Four experienced research assistants (RAs) were engaged and trained for four days on the data collection exercise with a developed training manual comprising data collection instruments on context, tool contents, and protocols for conducting successful interviews, including ethics and informed consent. Each training session lasted four hours. The RAs engaged in role plays to ensure that they all understood the questions being asked in the various instruments. The selection of RAs was based on their specialty in disability studies and experience with data collection.

### 2.5. Phase One: Quantitative Phase

Using the Lwanga, Lemeshow, and WHO [35] formula, an effective sample size of 399 was obtained and considered sufficient for the quantitative phase of the study. A two-stage health-facility-linked approach was used to select the HPs. In the first stage, the study area was grouped into rural (Offinso North) and urban (Kumasi Metropolis) areas and included all the major government health facilities. In the second stage, 399 HPs were recruited based on the inclusion criteria and recommendations for health facility surveys by Turner, Angeles, Tsui, Wilkinson, and Magnani [36] to ensure a wide geographical spread of HPs.

#### 2.5.1. Survey Instrument

The data collection instrument was developed from a validated questionnaire [37] and previous literature [4,14,15]. The instrument had various sections, but only three main sections were used for this study, comprising background characteristics, attitude towards PwDs, and enablers and barriers to SRH delivery. The background characteristics section elicited information on sociodemographic and work-related characteristics such as age, sex, marital status, highest qualification, number of years in practice, and the type of health facility. The attitude towards PwDs section was adapted from the Attitude Towards Disability Scale (ATDS) [37], a 16-item instrument with four sub-scales—inclusion, discrimination, prospects, and gains ([37], p. 872). Each sub-scale has four questions measured on a five points Likert scale (Strongly agree = 1 to Strongly disagree = 5). The total score ranged from 16 to 80. Higher mean scores for each domain were indicative of better inclusion, less discrimination, more gains, and better prospects, respectively. The instrument has been used in various settings by researchers and its internal consistency reliability using Cronbach alpha has varied from 0.70 to 0.92. In this current study, the overall reliability was 0.71 [38,39,40].

The instrument was pre-tested at the Nkawie and Nkenkaasu Health Centres among 20 HPs before full data collection. Permission was obtained at the point of data collection with informed consent. The average duration to complete an interview was 15 min. After this, respondents were also asked to indicate their willingness to participate in the qualitative phase of the project, and those who agreed were asked to provide their contact details. The pre-testing showed that all the questions were clear and unambiguous.

#### 2.5.2. Data Analysis

The quantitative data were analysed with Stata version 14 for Mac OS (Stata Corporation, College Station, TX, USA). Descriptive statistics for the sociodemographic characteristics and the barriers to SRH service provisions to PwDs were reported using frequencies and percentages. The continuous score of the ATDS was set as the dependent variable, while the background characteristics were the independent variables. The independent samples t-test or one-way analysis of variance (ANOVA) was used as appropriate to assess the relationship between ATD and the independent variables. In all the analyses, statistical significance was set at *p*  <  0.05.

### 2.6. Phase Two: Qualitative Phase

This part of the study was an opportunity to gain a deeper understanding of some of the issues that were found in the quantitative phase, and 25 HPs were purposively selected for these in-depth interviews. One female RA who was part of the first phase and the lead author (A.S.) conducted all the interviews. The RA was trained using the training manual on appropriate interview techniques, such as appropriate community entry (access to health facility and HPs), interviewing skills, consent seeking, probing, avoiding leading questions, appropriate note-taking, and the use of an audio recorder. The training was also complemented by role plays, where A.S. and the RA played the roles of an interviewer and interviewee interchangeably to ensure that the questions were well understood. Prior to the data collection, the in-depth interview guide was pre-tested among 5 HPs (1 doctor, 2 midwives, and 2 nurses) at the Kumasi South Hospital and Akumadan Health Centre. These participants were excluded from the main study.

After the pre-testing, the questions were clear and sought to provide answers to achieve the objectives of the study. All the interviews were conducted face-to-face at the identified hospital locations that were deemed safe and convenient for the RAs and participants. All the interviews were audio recorded with both verbal and written permission from the participants. Notes were also taken to capture non-verbal cues. The average interview duration ranged from 48 to 70 min. At the 23rd participant, data saturation was reached. However, two nurses who previously expressed interest were also interviewed to confirm that, indeed, new themes and concepts were not emerging. The RAs informed the participants that they would be contacted as and when the need arose. However, this was not necessary since all the issues discussed were clear and exhaustive.

#### Qualitative Data Analysis

Audio recordings of the in-depth interviews were transcribed verbatim by AS and the RA. The transcripts were crosschecked with the audio recordings to ensure that the original recorded interviews and the transcribed version were in tandem with each other. Inductive thematic analysis was employed ([41], p. 87) using NVivo version 12 (QSR International, Ltd., Daresbury Cheshire, UK). All the data transcripts were read while carefully taking notes and highlighting key and salient issues. The next stage of “generating initial codes” was carried out inductively by A.S. with expert inputs from K.M.R. and B.S.M.-A., who frequently met to discuss structure and direction. The third stage of “searching for themes”, A.S., K.M.R., and B.S.M.-A. highlighted and grouped some aspects of the data into similar themes or key topical areas before “reviewing the themes” in the fourth stage by regrouping and establishing linkages between themes. A.S., K.M.R., and B.S.M.-A. identified, discussed, and agreed on the relevant quotes to the themes, while T.I.E. and A.E.O.M.A. confirmed, defined, and named the reviewed themes. Finally, verbatim quotes were used to represent the various themes that emerged from the data with their relevant demographic information (sex, age, type of health worker, and district).

## 3. Results

### 3.1. Phase One: Quantitative Findings

#### 3.1.1. Sociodemographic Characteristics of Respondents

Table 1 presents the sociodemographic characteristics of the 399 respondents. The mean age was 29.6 ± 5.4, with a range of 19 to 57 years. The majority (37.1%) of the respondents were aged 25–29, and 18.1% were below 25 years. Most (81.4%) were female. Slightly more than half (53.6%) were never married. About 85% of the respondents were Akans and 91.2% were Christians, while 82% indicated they had received education about PwDs. More than half (54.4%) of the respondents were nurses, and 62.2% had diploma degrees. 

#### 3.1.2. Factors Influencing the Attitude of Healthcare Providers towards Persons with Disabilities

Table 2 shows the results on the distribution of total attitude score and subscale scores by background characteristics. Overall ATD score was 46.9 ± 6.8 ranging from a lowest score of 23 to a maximum score of 69 out of the total score of 80. Those aged 35+ had the highest mean score of 47.3 ± 6.9. Marital status significantly differed across the various groups. For example, those who were never married had the lowest attitude score (46.0 ± 6.9; *p* = 0.04), indicating that they held more negative attitude towards PwDs. Compared to those working in smaller health centres (44.1 ± 5.8), HPs working in district hospitals (47.6 ± 6.7; *p* = 0.001) had more positive attitudes towards PwDs and were less discriminatory (9.9 ± 2.7; *p* = 0.01). Additionally, HPs in Kumasi metropolis (10.2 ± 2.9; *p* = 0.04) were more positive towards PwD’s inclusion in social activities compared to those in rural areas (9.5 ± 2.5). In relation to positive gains, those who have ever received education about PwDs (12.6 ± 3.2; *p* = 0.04), and those working in district hospitals (12.9 ± 3.1; *p* = 0.001) had more positive attitudes compared to their respective counterparts. Interestingly, for PwDs’ prospects, male HPs (15.9; ±2.9) had more positive attitude scores than females (15.0; ±2.9, *p* = 0.01).

#### 3.1.3. Healthcare Providers’ Perception of the Factors Hindering the Successful Delivery of Sexual and Reproductive Health Services to Persons with Disabilities

**Figure 3:**
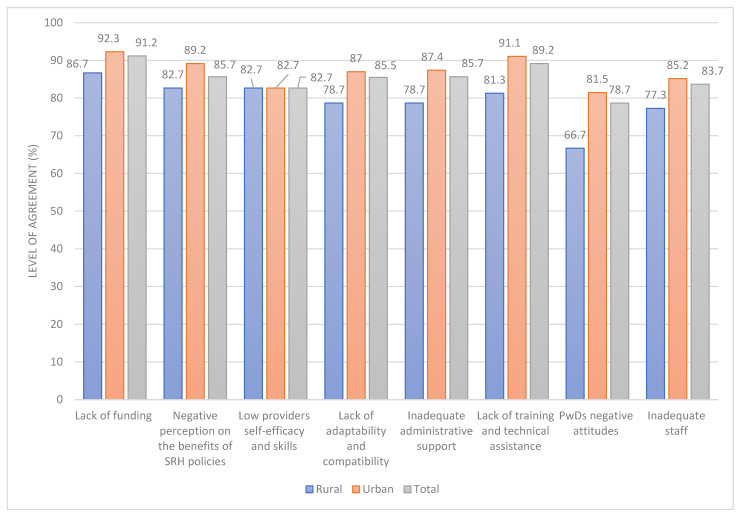
Factors influencing successful delivery of sexual and reproductive health services to persons with disabilities.

Figure 3 presents the results of HPs’ perception of factors hindering the delivery of SRH services to PwDs. The majority (92.3%) of HPs in the urban areas indicated that lack of funding to support SRH services delivery was a major barrier. Lack of training and technical assistance to provide services to PwDs (91.1%), negative perception (89.2%) of the benefits of the SRH policy or programs to PwDs, and inadequate managerial/supervisory/administrative support (87.4%) were the top four barriers indicated by HPs in the urban area. Likewise, the top four barriers for HPs in rural areas were lack of funding (86.7%), low provider self-efficacy and skills to deliver SRH services (82.7%), negative perception (82.7%) of the benefits of the SRH policy or programs to PwDs, and lack of training and technical assistance (81.3%) (See Figure 3).

### 3.2. Phase Two: Qualitative Findings

**Figure 4:**
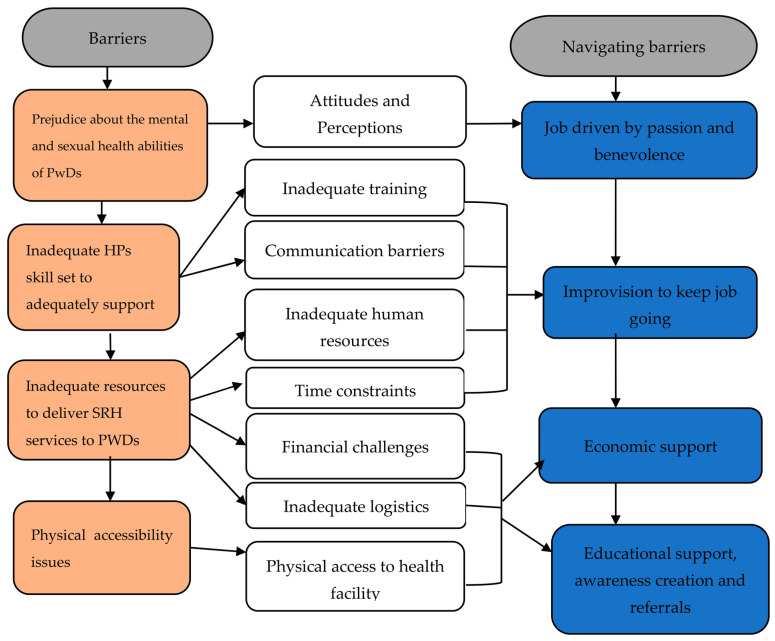
Themes emerging from the data and their interrelationships.

In total, 25 participants (16 females and 9 males) participated in the qualitative phase of the study. Their ages ranged from 28 to 60 years; 9 were from the Offinso North District and 16 were from the Kumasi metropolis. There were eight overarching themes that emerged. Four of them (prejudice about the mental and sexual health ability of PwDs, an inadequate HPs’ skill set to adequately support PwDs, inadequate resources to deliver SRH services to PwDs, and physical accessibility issues) were related to the challenges they faced in the process of delivering SRH services to PwDs, while the other four (compassion and benevolence towards PwDs, improvision, economic and educational support, awareness creation, and referrals) were strategies they used to overcome those challenges. Figure 4 shows the diagrammatic representation of the themes and their interrelationships.

#### 3.2.1. Prejudice and Misconceptions about the Mental and Sexual Health Ability of Persons with Disabilities

In terms of prejudice and misconceptions, some of the HPs indicated that they generally discriminated against PwDs because they felt that they were difficult to deal with, hard to explain things to, and the perception that they were unable to engage in sexual activities and the feeling that if they over engaged with PwDs, their colleagues would laugh at them.

Healthcare providers’ attitudes and perceptions about persons with disabilities:

“*We [healthcare providers] discriminate against them all the time even among my colleagues at work. The moment they see you talking to someone with a disability, they turn to discriminate against you for speaking with a person with a disability*”.

(Midwife, Kumasi, Female, 56 years)

“*I don’t think a lot of people can boldly walk to a pharmacy shop to buy a condom. How much more someone with a disability. People have this perception that sex should not even be part of the life of persons with disabilities at all*”.

(Nurse, Kumasi, Female, 46 years)

#### 3.2.2. Inadequate Healthcare Providers’ Skill Set to Adequately Support Persons with Disabilities

Participants also reported on their limited skill set to adequately provide SRH services to PwDs which included limited training and a lack of communication skills. The successful delivery of SRH services to PwDs by participants was influenced by these factors.

##### Inadequate Training

The majority of the participants expressed deficiencies in skill sets to adequately address PwDs SRH issues. In most cases, they had never received training on how to interact or support PwDs. They saw this as a significant barrier to appropriately engaging with PwDs.

“*I haven’t received any training throughout my 15 years of work in this facility on how to care for persons with disabilities*”.

(Nurse, Kumasi, Female, 46 years)

“*…No, I haven’t received any training, but along the line, they conduct workshop for us and sometimes an issue about disability can come for us discuss it but we don’t go through proper training on how to support PwDs*”.

(Nurse, Kumasi, Female, 59 years)

##### Communication Challenges

The participants also expressed that the lack of training impacted on their communication with the PwDs. Specifically, participants reported their inability to understand and communicate with hearing-impaired PwDs using sign language, which raised issues of privacy and confidentiality.

“*Since I didn’t learn how to interpret sign language and communicate through sign language, it is sometimes challenging for me to communicate with someone who is deaf [referring to hearing impaired]. So, in the case of my client, the mother was the interpreter, and this can be challenging if the client wants you to keep some secret or doesn’t want her mother to know certain things. In short, it breaches confidentiality*”.

(Doctor, Kumasi, Female, 32 years)

#### 3.2.3. Inadequate Resources to Deliver SRH Services to PwDs

The third theme about the challenges HPs faced in their provision of SRH services that emerged from the data was inadequate resources to deliver SRH services to PwDs. Four key issues were discussed; these were inadequate human resources, time constraints, financial challenges, and inadequate logistics.

##### Inadequate Human Resources

Another factor that significantly affected HPs adequately providing SRH services to PwDs was inadequate human resources to cater for the needs of PwDs.

“*For those with disabilities, we don’t have workers to handle them. Unlike those without disabilities, we have enough health care providers to provide sexual and reproductive health services to them*”.

(Nurse, Offinso, Female, 37 years)

##### Time Constraints

In addition, some participants indicated how the amount of time spent during the care of PwDs was a significant barrier that prevented them from providing adequate SRH services to PwDs.

“*The only thing is that the amount of time that I will spend on the PwD will be more than I have spent on persons without disabilities. I have to spend a lot of time asking questions and explaining things to them. We don’t give them so much information at once. If you do that, you will confuse them*”.

(Physician Assistant, Offinso, Male, 45 years)

“*And also, some of the PwDs come without relatives so that means you have to leave your post to help them find a means of transportation back to wherever they came from*”.

(Nurse, Offinso, Male, 28 years)

##### Financial Challenges

Almost all the participants reported how financial challenges affected their delivery of SRH services to PWDs. This impacted the ability of PwDs to access SRH services because they could not afford some prescribed medications. In addition, the participants discussed how the ineffectiveness of the national health insurance scheme negatively impacted access to SRH services by PwDs.

“*The challenge is that because of the financial constraints of PwDs, they refuse to buy medications that are prescribed to them. Due to this within the shortest possible time, they will report to the facility with the same problem again*”.

(Nurse, Offinso, Male, 34 years)

“*There are medications that are covered by the national health insurance scheme and those that are not. Moreover, there are certain medications that will be covered by the health insurance in the hospital [Higher level] but in health centres [lower level] like our facility, it is not covered. So, that is where the challenge lies*”.

(Midwife, Offinso, Female, 35 years)

##### Inadequate Logistics

Furthermore, the unavailability of special equipment to aid SRH delivery to PwDs was another resource constraint the HPs discussed.

“*We don’t have special equipment to use for PwDs. We just use the same equipment and logistics that we use for persons without disabilities. But sometimes due to their special nature, we need some beds that are more adjustable to meet their needs. Just imagine, currently, we do not even have surgical gloves for deliveries*”.

(Midwife, Kumasi, Female, 30 years)

#### 3.2.4. Accessibility Challenges of Persons with Disabilities in Accessing SRH Services

The fourth theme that emerged from the data on the challenges HPs face in providing SRH services to PwDs was accessibility. The major accessibility issue the participants discussed was PwDs’ physical inaccessibility to health facilities. The participants described how the unfriendly nature of some health facilities impacted on their successful delivery of SRH services to PwDs.

“*For those with disabilities I think they are left behind… Looking at our buildings and how they’ve been arranged, from stairs to everything you can see that it’s not favourable to them and at times even coming to the facility is also a big challenge because we don’t have any equipment to take care of them*”.

(Midwife, Kumasi, Female, 56 years)

“*Our consulting rooms are not well structured for patients with disabilities. Like the patients with disabilities using wheelchairs, we don’t even have walkways for them*”.

(Doctor, Kumasi, Female, 32 years)

#### 3.2.5. Strategies Employed to Circumnavigate Barriers

Four themes were identified in relation to the participant’s strategies in overcoming some of the bottlenecks encountered in SRH services delivery to PwDs: (a) the job is a calling driven by compassion and benevolence towards PwDs; (b) improvision to keep the job going; (c) supporting financial needs of PwDs; and (d) educational support, awareness creation and appropriate referrals to avoid complications.

##### The Job Is a Calling Driven by Compassion and Benevolence towards Persons with Disabilities

Despite participants reporting that they had not received adequate training to support PwDs, most participants expressed their eagerness to assist PwDs who accessed their health facilities. As a result, some HPs go over and beyond their call of duty to ensure that they provide SRH services to PwDs.

“*Well, our job is mainly driven by compassion. It is a calling. So, if you do not make up your mind to do it, you might be disheartened by the attitudes of some of the clients because the process of childbirth is stressful and causes them to behave in an unfriendly manner*”.

(Kumasi, Midwife, Female, 35 years)

##### Improvision to Keep the Job Going

Another strategy the participants discussed to overcome the challenges they face in their quest to deliver SRH services to PwDs was improvision. Some of the participants leveraged technological innovations to deliver SRH services to PwDs.

“*There is this client that I had. She had a hearing problem (referring to hearing impaired). What we do is, she writes on her phone and she shows it to me, I read, I also type my questions on my phone and I show it to her, she reads. So that’s how we went about it till she delivered. So in such instances if you meet someone who is unable to read and write it becomes a bit challenging*”.

(Kumasi, Doctor, Female, 32 years)

##### Supporting the Financial Needs of Persons with Disabilities

Furthermore, the participants reported personal and institutional level financial support as another strategy they employed to overcome the challenges. Some of the health facilities had set up special units to ensure financial support for PwDs.

“*Sometimes, we have to use our own money as health providers to support the financial needs of the PwDs. I remember there was a physically challenged woman who came here and she was feeling so bad that she decided to lie on the bed and sleep. Her caregiver just rushed in shouting at the woman and asking her whether she thinks they can afford it. So, we decided to waive whatever that the woman would have paid because looking at the situation, there is no way she could have afforded it*”.

(Midwife, Kumasi, Female, 56 years)

“*Those who are not able to pay for their hospitals bills such as labs, admission, and consultations, we have a unit here that takes care of that. So, we just refer them to that unit for them to handle it*”.

(Doctor, Kumasi, Female, 32 years)

##### Educational Support, Awareness Creation, and Appropriate Referrals to Avoid Complications

In addition, participants discussed how they provided educational support to PwDs to seek financial assistance. Some of the participants also discussed the role of referrals in the management and prevention of complications.

“*The situation is compounded for PwDs as most of them are unable to work. So, in such situations, we try to resolve issues of finances by encouraging PwDs to register and enrol onto the national health insurance scheme. We educate them about the importance of registering for the health insurance*”.

(Nurse, Offinso, Male, 34 years)

“*If you get a case that is beyond you. You need to do an assessment and then refer appropriately to avoid any further complications*”.

(Midwife, Kumasi, 30 years)

“*For cases that we cannot handle, we refer to the hospital because our facility is a health centre. We first communicate with the patient and then call the referral facility to inform them that we are referring a case. When we call the referral centre and they are unreachable, we allow one of our staff to accompany the PWD to the referral centre. If it is antenatal and it is not an emergency, then you can allow her to go by herself*”.

(Midwife, Offinso, 35 years)

#### 3.2.6. Triangulation of Study Findings

A synthesis of the results from the quantitative and qualitative phases was completed. A summary of the combined findings and representative participant quotes are presented in Table 3.

## 4. Discussion

The study assessed the attitudes of HPs towards PwDs and enablers and barriers towards SRH services delivery to PwDs in Ghana’s Ashanti Region. Previous studies have shown that the attitude of HPs towards PwDs is a reflection of how the general society sees PwDs [3,4]. In the present study, HPs had a relatively positive attitude towards PwDS with a mean score of 46 (range 23–69). This finding is similar to previous studies in some HICs, such as the USA [8] and Canada [9], and a systematic review by Satchidanand et al. [42]. Nonetheless, our current findings are different from what was found in an LMIC, Nepal [4] and some HICs such as the USA [43] and Australia [44]. In this present study, HPs had low scores in the discrimination domain, indicating a discriminatory attitude towards PwDs. From the foregoing contrasting results, the attitudes of HPs towards PwDs in LMICs and HICs are inconclusive. The possible reasons for the relatively positive attitude of HPs towards PwDs in this current study might be influenced by the majority (82%) of the respondents indicating that they had heard about disability-related issues either in school or from the media. It is noteworthy that the comparison of the studies warrants caution since varied scales were used to measure the attitudes of HPs towards PwDs.

Similar to previous studies and the conceptual framework, it was found that the personal characteristics of HPs influenced their attitudes towards PwDs, however, the results are generally inconclusive [4,10,11,12,13]. In this present study, males had significantly higher attitude scores on the prospects domain compared to females. The current findings are similar to those of Devkota et al. [4] and Ismail et al. [13] in Nepal and Malaysia, respectively. Nonetheless, the findings are different from other studies [10,11,12] that reported women as having higher positive attitudes than men. The general notion is that women are more empathetic, which translates to their positive attitudes toward PwDs; however, this was not the case for this study. The possible explanation for this contrasting finding is the variation in the level of education of males and females in this study, in which males had a higher level of education compared to females. This highlights the importance of adequate training for HPs to facilitate equitable and effective SRH services to PwDs.

HPs in the urban areas (Kumasi) had significantly higher mean scores than those in the rural areas (Offinso) in the inclusion domain. Similar findings were observed among HPs working in higher-level hospitals (Regional and Teaching hospitals) compared to those in rural health centres (lower level). This means that HPs in urban areas accepted the inclusion of PwDs in social issues. The probable reason for this finding is that those in urban areas are more exposed to educational content on disability-related issues and are also more likely to attend workshops compared to those in rural areas. In addition, there are deep-rooted sociocultural misconceptions about disability in rural areas in Ghana [45,46]. This calls for the intensification of anti-discrimination campaigns and education on the inclusion of PwDs in various rural societies and the general Ghanaian society.

The findings also showed that those who have ever received any information on disability issues had significant higher attitude scores compared to those without any information. This was also reflected in the educational status of the HPs. For example, those with Masters and Bachelor’s degrees had significantly higher scores in the prospects domain compared to certified nurses. This highlights the importance of education and training on attitude formation and change of HPs towards PwDs. The possible explanation is that those with a higher level of education are exposed to disability-related issues during their training. Some of them might have received training on disability at different times at the various levels of their education. Similar findings have been reported in the USA among nurses who attained more positive attitude scores when they participated in a before and after study on disability education programme [47]. However, Devkota et al. [4] did not find any variation in attitude scores among HPs in Nepal who received training and those who did no. They argued that short-term training had a lower probability of changing the attitudes of HPs positively and called for long-term education and training.

The study also provides insights into the experiences of HPs in providing SRH services to PwDs in the Kumasi metropolis and Offinso North District of Ghana. Their experiences included the challenges they encountered and the strategies used to circumvent them. Overall, the findings indicate that HPs face numerous obstacles in providing services to PwDs. This is consistent with several previous studies which have found that the main obstacles to providing services to PwDs include prejudice, misconceptions, and difficulties working with PwDs, inadequate skillset to care for PwDs, inadequate resources, and inaccessibility [2,5,48,49,50,51,52]. Importantly, this means that the challenges facing HPs in their delivery of SRH services to PwDs are a systemic issue in Ghana. Yet, PwDs frequently face the brunt of the entire systemic failure [48], specifically, with an inadequate skill set to care for PwDs, where participants mentioned that they lacked the necessary training and knowledge on disability issues. It is noteworthy that from the quantitative phase, HPs indicated more awareness about disability; however, this might not necessarily translate to knowledge and ability to care. This is one of the major challenges in service provision to PwDs globally [2,5,53]. Participants believed that the lack of disability training on the job or during their study greatly hampered their care to PwDs, which subsequently affected their ability to communicate with PwDs and limited the time spent on providing SRH services to PwDs. Previous studies in the USA [50], Nepal [4], Bangladesh [51], and Ghana [16,48] have also found similar results. For example, Acheampong et al. [16] indicated that HPs find it challenging to communicate with the hearing impaired and this hampers their quality of care.

In this present study, the participants indicated that challenges with communication raised ethical and confidentiality issues because some health issues were so sensitive that PwDs did not want to share such information with their caregivers or any third party, although there were instances where HPs had to rely on third parties to interpret for PwDs. The reliance of HPs on third parties meant that the privacy and rights of their patients were violated because others learned about their health conditions. The care provided to PwDs may be ineffective if the HPs do not understand their issues. The ability of PwDs to use SRH in the future could be subsequently impacted. Hence, it is imperative to employ certified sign language interpreters in the various health facilities who can attend to the needs of PwDs as and when necessary. If the provisions of the 2006 Ghana Disability Act 715 [54] are to be achieved and the rights to privacy of PwDs respected and maintained, this necessitates the intensification of training for HPs in the use of sign language and disability issues in general. It is therefore advised that the Ministries of Education, Health, and the Ghana Health Service collaborate closely with the various educational institutions that train healthcare professionals to review curricula and incorporate fundamental knowledge of disability issues, including strengthening the use of sign language training for health communication, so that the professionals will have a comprehensive understanding of disability issues.

Inadequate resources leading to delays in service provision and time constraints were other major barriers raised by HPs. Similar to our current research, the scarcity of providers and services is a global issue and has a detrimental impact on the ability of PwDs to receive SRH care [48,51]. Dassah et al. [48], for instance, found that the lack of resources and providers in rural Northern Ghana is a major challenge. Similar to previous studies [15,16,50], it was reported that due to the special nature of the care and needs of PwDs, some of the facilities with limited numbers of HPs found it challenging to find ample time for PwDs. Some indicated that the amount of time they spent on PwDs caused a lot of pressure since they had many clients to take care of; this could also lead to staff burnout and its resultant adverse effects on care delivery. It is, therefore, important to increase the number of HPs in various health facilities within the Ashanti region to promote equity in healthcare accessibility, as argued by Dassah et al. [48] and the World Report on Disability [55]; this could further aid the achievement of the Universal Health Coverage agenda [56].

To overcome the challenge of inadequate skill sets, participants adopted various strategies that included improvising and self-sacrifices by relying on text messaging among clients and supporting clients financially to keep the job going. This also shows the opportunities that the use of technology can present to reduce challenges in SRH healthcare delivery to PwDs [57]. The emphasis on self-sacrifice is similar to earlier studies on caring for PwDs [48,58]. Dassah et al. [48] argued that one of the fundamental concepts of health professional organisations and agencies globally is providers’ self-sacrifice. For instance, the fundamental principles of professional values (such as self-sacrifice) established by Ghana Health Services may include exhibiting a high sense of dedication to responsibility for preserving the dignity and interests of patients without discrimination [59]. This finding can also be discussed within the context of the charity model of disability [60], which argues that some people do good activities for PwDs to get blessings from God, as some of the participants indicated “*You care for PwDs and they will then bless you*”. It is, therefore, important to leverage the compassion and benevolence of HPs to intensify training opportunities that can improve their attitudes towards PwDs and SRH delivery to PwDs. Institutional award systems for such HPs could also motivate others to adopt such traits. Similar findings on improvision were reported by Dassah et al. [48] in rural Northern Ghana.

Another major finding from both the quantitative and qualitative phases was inadequate resources. Specifically, HPs indicated encountering financial challenges and unavailability of logistics to meet the needs of PwDs. Even though Ghana’s health insurance scheme was implemented to lower the financial barriers to access, there are still access disparities between the general public and the PwDs [61]. For example, the participants’ experiences point to the ineffectiveness of the national health insurance scheme to provide financial support to PwDs to access SRH healthcare. Although some of the participants indicated how it has been instrumental in SRH health accessibility among PwDs previously, the scheme has been ineffective in recent times since most essential drugs are not covered. As a result, PwDs bore the cost of medications, calling for the need to strengthen the national health insurance scheme for PwDs to benefit more from it and to improve their SRH healthcare access and outcomes.

In this regard, it was also found that physical inaccessibility to health facilities in terms of transportation and facility structures was another challenge that HPs indicated in both the quantitative and qualitative phases. As such, PwDs who accessed the health facility without a support person meant that HPs must leave other clients and escort them to get means of transport home before they could come back to continue their duties. This finding has been reported in previous systematic reviews [2,5,53], other studies in Ghana [5,16,48], and in other countries such as Zimbabwe [62] and Nepal [4]. Relatedly, most participants indicated how inadequate logistics, such as weighing scales and adjustable beds, serve as a major challenge to providing service for PwDs. Similar findings have been reported by Mitra et al. [50] in the USA and Dassah et al. [48] in Ghana.

Discussing the findings within the health outcomes model (Figure 1), Mitchell et al. [25] and Radwin [26] point to the interplay between system characteristics, the nature of health delivery, and how a complicated interaction between supply and demand affects access to SRH healthcare among PwDs. For instance, it was found that barriers to SRH health delivery resulted from the healthcare system’s incapacity to cater to the healthcare needs of PwDs in the form of inadequate HPs, unfriendly health infrastructure, and inadequate logistics. This pattern is also consistent with a previous study in Ghana that there is a mismatch between the supply and demand of HPs [48].

However, several of the participants described how they had to overcome financial challenges, logistics, and physical access to health facility obstacles by providing economic support, educational support on available opportunities, awareness creation, and referring clients. Specifically, some of the participants shared how they used their own money to take care of clients’ medical bills or pleaded with some of their colleagues to waive the medical bills for PwDs. Participants also indicated how some health facilities had designated units to support the financial needs of PwDs who were unable to pay their medical bills. In addition, some of the participants indicated that they always had to educate PwDs who had not enrolled in the national health insurance scheme to do so. Moreso, some of the HPs indicated how they referred patients to avoid further complications, especially those in labour. Nevertheless, research has shown that financial difficulties (i.e., costs associated with care and transportation) and a long distance from facilities, along with indecipherable risks, frequently discouraged referral uptake for PwDs to medical facilities [63]. The study’s participants took particular note of this issue and remarked that there were instances where they referred PwDs, but they refused the referral with the excuse that they either had no money or were afraid to receive poor care at different facilities.

### 4.1. Implications for Policy and Practice

The study findings have various implications for policy and practice. Several international and national documents have stipulated the need to ensure SRH accessibility for all, including PwDs. For example, the UNCRPwD’s Article 25 re-enforces the rights of PwDs to attain the highest standard of healthcare without discrimination and that various nations should provide healthcare taking into consideration the needs of PwDs [64,65]. The sustainable development goal (SDG) No. 3 also seeks to ensure universal access to healthcare and argues that the inclusion of PwDs in every aspect of life is crucial for sustainable development [66]. The SDGs also emphasises the need for Universal Health Coverage, which will increase access to healthcare for all people [56]. This encompasses all subgroups of the population, including PwDs. The 2006 persons with disability act 715 of Ghana also mandates health providers to provide services to PwDs. The WHO disability action plan 2014–2021 also indicated the need for access to services to achieve health and well-being, and human rights for PwDs. According to some experts, if healthcare initiatives fail to include PwDs, it demonstrates that they are unproductive [67]. Despite this, the attainment of these provisions might be problematic since HPs face numerous barriers in the delivery of SRH services to PwDs. It is, therefore, necessary for the government and health administrators to ensure that training to eliminate prejudice and misconceptions about PwDs for all HPs is mandatory. To facilitate better communication between HPs and PwDs, it is recommended that the employment of certified sign language interpreters be expanded. This will strengthen SRH care provision and accessibility.

### 4.2. Strengths and Limitations

There are various strengths of the paper that are worth mentioning. This is the first study using a mixed method design to assess HPs’ attitudes and the enablers and barriers towards the delivery of SRH services for PwDs in the Ashanti region of Ghana. Kroll et al. [68] explained the importance of adopting the mixed methods design in disability and rehabilitation studies. They argued that “*the combination of both methods is well suited for evaluation studies where the qualitative component may provide answers to “why things work or not” and the quantitative component may measure to what extent a program is successful*” (p. 112). Therefore, this current study has shown the challenges HPs encounter and how they are able to overcome those challenges in providing SRH services to PwDs. Second, the quantitative phase had a relatively large sample size. Third, the use of a validated scale (ATDS) that is widely used in diverse contexts to measure attitude is also a strength of this study. The study also considered diverse groups of HPs in both rural and urban areas. Despite these, there is the possibility of bias due to social desirability since most of the issues were self-reported, and the participants for the qualitative phase were those who expressed further interest in the issues under study.

## 5. Conclusions

HPs had relatively positive attitudes towards PwDs, and this varied across sub-scales of the ATDS and sociodemographic characteristics. HPs also faced several barriers in their delivery of services to PwDs, and these were confirmed in the qualitative phase of the study. However, they navigated these challenges through compassion and benevolence towards PwDs, improvision, economic support, educational support, awareness creation, and patient referrals. Mandatory training of HPs is recommended to ensure improved SRH service delivery to PwDs. Future research could explore the perceptions and coping strategies of PwDs.

## Figures and Tables

**Table 1:** Sociodemographic characteristics of respondents.

Variable	Frequency	Percentage
**Age (Mean ± SD)**	29.6 ± 5.4	
Below 25	72	18.1
25–29	148	37.1
30–34	105	26.3
35+	74	18.5
**Sex**		
Female	325	81.4
Male	74	18.6
**District**		
Offinso (Rural)	75	18.8
Kumasi (Urban)	324	81.2
**Marital status**		
Never Married	214	53.6
Married	163	40.9
Cohabiting/Widowed/Divorced	22	5.5
**Ethnicity**		
Akan	338	84.7
Mole-Dagbani	29	7.3
Ewe/Ga-Adangbe	22	5.5
Other **	10	2.5
**Religion**		
Christian	364	91.2
Non-Christian (Muslim, No religion)	35	8.8
**Education about PwDs**		
No	70	17. 5
Yes	329	82. 5
**Categories of Health Workers**		
Doctor/Physician Assistant	32	8.0
Midwife	150	37.6
Nurse	217	54.4
**Years in Service (years)**		
Below 5 years	277	69.4
5–9	70	17.5
10 and above	52	13.0
**Highest Qualification**		
Certificate Nurse	36	9.0
Diploma	248	62.2
Bachelor’s Degree	95	23.8
Masters/Doctorate	20	5.0
**Type of Facility**		
Health Centre (lower level)	15	3.8
Hospital (intermediate)	288	72.2
Teaching Hospital (higher level)	96	24.0
**Total**	399	100.00

PwDs: persons with disabilities; SD: standard deviation; ** Other: Guan, Fulani, Yoruba.

**Table 2:** Factors influencing the attitude of healthcare providers towards persons with disabilities.

Variable	Total Attitude Score Range (16–69)		Inclusion Score (Range 5–20)		Discrimination Score (Range 5–20)		Gains Score (Range 5–20)		Prospects Score (Range 5–20)	
	Mean(SD) 46.9(6.8)	*p*-Value	Mean(SD) 9.6(2.6)	*p*-Value	Mean(SD) 9.6(2.8)	*p*-Value	Mean(SD) 12.5(3.2)	*p*-Value	Mean(SD) 15.1(3.0)	*p*-Value
**Age**		0.78		0.27		0.75		0.80		0.34
Below 25	46.7(6.1)		10.2(2.5)		9.5(2.5)		12.1(2.9)		14.9(2.5)	
25–29	46.5(7.0)		9.6(2.4)		9.6(2.8)		12.4(3.1)		15.0(3.1)	
30–34	47.2(6.5)		9.5(2.7)		9.8(2.7)		12.5(2.9)		15.3(2.7)	
35+	47.3(6.9)		9.6(2.9)		9.4(3.1)		12.7(3.9)		15.6(2.9)	
**Gender**		0.84		0.89		0.15		0.21		0.01 *
Female	46.9(6.9)		9.7(2.6)		9.7(2.8)		12.6(3.2)		15.0(2.9)	
Male	46.7(6.6)		9.6(2.5)		9.2(2.7)		12.0(3.1)		15.9(2.9)	
**District**		0.80		0.04		0.83		0.36		0.96
Offinso (Rural)	46.8(6.9)		9.5(2.5)		9.6(2.8)		12.5(3.2)		15.2(2.9)	
Kumasi (Urban)	47.1(6.4)		10.2(2.9)		9.5(2.9)		12.2(3.1)		15.2(2.6)	
**Highest qualification**		0.96		0.75		0.04		0.16		0.01 *
Certificate	47.0(7.0)		10.1(2.6)		10.5(3.3)		11.9(2.8)		14.6(2.8)	
Bachelor’s Degree	47.9(6.4)		9.7(2.5)		9.3(2.7)		12.3(3.3)		15.7(2.5)	
Diploma	46.9(7.0)		9.6(2.6)		9.7(2.8		12.7(3.1)		14.9(3.0)	
Masters	46.2(6.1)		9.8(2.8)		8.5(2.8)				16.6(2.3)	
**Number of years in practice**		0.94		0.74		0.63		0.77		0.64
<5 Years	46.9(6.6)		9.7(2.6)		9.7(2.7)		12.4(3.0)		15.1(2.9)	
5–9 years	46.9(7.6)		9.4(2.9)		9.3(2.9)		12.7(3.3)		15.4(2.8)	
10+	46.6(7.0)		9.6(2.6)		9.5(3.5)		12.6(3.9)		15.0(3.0)	
**Marital status**		0.04 *		0.65		0.32		0.01 *		0.23
Never Married	46.0(6.9)		9.6(2.6)		9.4(2.7)		12.1(3.1)		14.9(2.7)	
Married	47.8(6.5)		9.7(2.7)		9.8(3.0)		12.8(3.2)		15.4(3.0)	
Cohabiting/ Widowed/Divorced	48.1(6.8)		9.2(2.7)		10.2(2.0)		13.7(3.6)		15.0(3.4)	
**Religion**		0.63		0.13		0.14		0.09		0.23
Christian	46.9(6.9)		9.6(2.6)		9.5(2.8)		12.5(3.2)		15.1(2.9)	
Non-Christian (Muslim, No religion)	47.3(6.0)		10.1(2.7)		10.3(2.7)		11.6(3.0)		15.3(2.4)	
**Type of health facility**		0.001 *		0.28		0.01 *		0.001 *		0.83
Health Centre	44.1(5.8)		10.5(2.4)		8.4(2.8)		10.4(3.8)		14.9(2.3)	
Hospital	47.6(6.7)		9.7(2.6)		9.9(2.7)		12.9(3.1)		15.2(2.9)	
Teaching Hospital	45.1(6.8)		9.4(2.7)		9.1(3.1)		11.6(3.0)		15.1(2.8)	
**Education about PwDS**		0.04		0.34		0.24		0.04 *		0.02 *
No	45.8(6.6)		9.9(2.9)		9.7(3.2)		11.8(3.2)		14.4(2.7)	
Yes	47.1(6.8)		9.6(2.6)		9.6(2.7)		12.6(3.2)		15.3(2.9)	
**Category of health worker**		0.60		0.40		0.05		0.06		0.003 *
Dr/PA	46.8(4.8)		10.3(2.4)		8.7(2.2)		11.2(3.0)		16.7(2.1)	
Midwife	46.5(6.8)		9.6(2.5)		9.5(2.9)		12.6(3.1)		14.8(2.8)	
Nurse	47.2(7.1)		9.6(2.7)		9.9(2.8)		12.6(3.3)		15.2(3.0)	
**Ethnicity**		0.97		0.99		0.94		0.05		0.28
Akan	46.9(6.8)		9.7(2.6)		9.6(2.7)		12.6(3.2)		15.1 (3.0)	
Mole-Dagbani	46.4(6.7)		9.6(2.9)		9.8(2.9)		11.6(3.0)		15.4 (2.1)	
Ewe/Ga	46.5(7.8)		9.7(2.1)		9.8(3.6)		11.5(3.2)		15.4 (2.9)	
Other **	46.6(5.1)		9.8(3.5)		9.3(3.3)		10.7(3.3)		16.8 (2.2)	

Significant results with *; TAS = total attitude score; ITS = inclusion total score; DTS = discrimination total score; GTS = gains total Score; PTS = Prospects Total score; SD = standard deviation; t = t-statistic; f = f-statistic; *p* = *p*-value; ** Other: Guan, Fulani, Yoruba.

**Table 3:** Triangulation of quantitative and qualitative findings.

Concept/Theme	Topic Areas	Quantitative Finding	Illustrative Qualitative Quote	Synthesis of Findings
Bottlenecks				
Prejudice about the mental and sexual health abilities of PwDs	HPs attitude and perceptions towards PwDs	Overall, HPs had just a little above average ATD score Overall ATD score = 46.9 ± 6.8.	“I don’t think a lot of people can boldly walk to a pharmacy shop to buy a condom. How much more [for] someone with a disability? People have this perception that sex should not even be part of the life of PwDs at all” (Nurse, Kumasi, Female, 46 years).	Even though HPs had slightly more than average ATD score, they scored low in the discrimination domain, indicating their negative attitude towards PwDs. This could explain some of their prejudice and misconceptions about the mental and sexual health ability of PwDs. It is, therefore, prudent to improve training opportunities and education of HPs about disability-related issues when in school and the need for refresher workshops when they are on the job.
		The discrimination score is also below average, indicating that HPs were more discriminatory towards PwDs. Discrimination score = 9.6 ± 2.8.	“I personally would not recommend that two PwDs marry themselves because they need helping hands. Most of them need support from others” (Doctor, Kumasi, Male, 28 years). “To be very honest, I am not comfortable anytime I am providing SRH services to them. Dealing with deaf and dumb (hearing impaired) is the most difficult thing…You cannot even seek consent to examine them sometimes…” (Midwife, Kumasi, Female, 30 years).	HPs reported how PwDs were stigmatised and discriminated by family members and society at large. However, some HPs were more hesitant to endorse marriage among PwDs, reflecting their discriminatory attitude towards them. Other HPs also indicated how they felt uneasy when providing care to PwDs. This could affect the quality of care provided. Educating and training HPs to have a more positive attitude towards PwDs will reduce their misconceptions and prejudice about them. It will also teach them appropriate ways to provide care to PwDs.
Inadequate HPs skill set to adequately support PwDs	Inadequate HPs training on disability-related issues and care	The majority (89.2%) of the HPs indicated a lack of training and technical assistance to provide SRH to PwDs as a barrier.	“That is a serious barrier. As we sit here, I cannot do sign language. My colleagues in this facility also lack the skills to provide care to PwDs using sign language or brails” (Doctor, Kumasi, Male, 28 years).	HPs training on disability-related issues and care is crucial to ensure adequate provision of SRH care to PwDs. The inability of HPs to understand some of the issues PwDs present can lead to misdiagnosis and dissatisfaction with the care provided. This could further impact on PwD’s future use of SRH services. Hence, it is pertinent to employ certified sign language interpreters in various health facilities to attend to the needs of hearing-impaired patients as and when necessary. This will facilitate SRH care provision.
	Communication barriers	HPs (82.7%) indicated the provider’s low self-efficacy and skills as one of the barriers to the delivery of SRH to PwDs.	“Since I didn’t learn how to interpret sign language and communicate through sign language, it is sometimes challenging for me to communicate with someone who is deaf. So, in the case of my client, the mother was the interpreter, and this can be challenging if the client wants you to keep some secret or doesn’t want her mother to know certain things. In short, it breaches confidentiality” (Doctor, Kumasi, Male, 32 years).	Since HPs were unable to communicate with PwDs in sign language, it sometimes breached the privacy and confidentiality of the conditions PwDs presented. Mostly, SRH issues are confidential. Therefore, some of the PwDs might be reluctant to share their conditions for HPs to get the full medical history to aid in diagnosis and treatment. HPs training on sign language should be strengthened in the various training schools by employing more sign language tutors.
Inadequate resources to deliver SRH services to PwDs	Inadequate human resources to deliver SRH services to PwDs	Participants (83.7%) indicated inadequate staff as one of the main barriers to the delivery of SRH services to PwDs.	“About staffing, we have serious issues. Can you imagine only one person is coordinating the project. Someone working with PwDs requires attention, time, and money” (NGO Official, Kumasi, Male, 60 years).	The HPs patient ratio has been a major issue in most health facilities in LMICs, including Ghana. The inadequate staff could lead to burnout and stress among the HPs. This will ultimately affect the quality of care they provide to their clients. Also, some PwDs might be discouraged from accessing SRH services at the health facilities due to the long waiting times they face when accessing SRH care. Therefore, government and health administrators need to increase the recruitment of HPs to adequately provide SRH care to PwDs.
	Financial challenges	Lack of funding to support SRH services delivery was a major barrier highlighted by the participants (91.2%).	“I think lack of funding is a major barrier. The government is not releasing a lot of money to support the implementation of the SRH policies” (Midwife, Kumasi, Female, 35 years).	Lack of funding could affect the purchase of essential medical supplies to aid in the delivery of SRH care to PwDs. It is important for Government to increase funding to health facilities to support the adequate provision of SRH services to PwDs.
	Time constraints in delivering SRH services to PwDs.	About 83% of the HPs reported that inadequate staff is a major challenge they encounter in SRH service provision since they need to spend more time caring for PWDs.	“I spend a lot of time on PWDs as I would spend on persons without disabilities. That makes our work difficult and stressful. But if they come with a relative, then it becomes a bit easy” (Midwife, Offinso North, Female, 35 years).	HPs indicated how support from PwDs’ relatives was crucial in their healthcare accessibility. This is because PwDs who did not come with aids needed to be supported to navigate the health system and this was sometimes undertaken by HPs. The time constraint, therefore, could impact on SRH care delivery negatively since HPs might be rushing to attend to other clients. The PwDs might also be dissatisfied with the services impacting their post-visit intentions. Essentially, in addition to the increase in healthcare providers, those who are newly recruited should be given on-the-job training to remind them of the need to provide required SRH services to PwDs.
	Inadequate logistics to provide SRH services to PwDs	Almost 86% reported that inadequate administrative support is a major barrier to the efficient delivery of SRH services to PWDs	“We don’t have enough logistics to support persons with disabilities since they need special care” (Doctor, Kumasi, Female, 32 years). “We do not have the logistics to cater for their needs. They access what people with no disabilities use, so there is nothing like special logistics for them which is sometimes challenging, you know” (Nurse, Kumasi, Female, 46 years).	The unavailability of logistics and equipment, such as adjustable beds to meet the needs of PwDs posed a serious challenge to HPs when providing services. This makes SRH provision to PwDs stressful for HPs. This can affect the quality of care to PwDs. Government and health facility managers should ensure that logistics and equipment are provided to meet the needs of PwDs to make it easy for them to access SRH care. It will also make it easy for the HPs to provide quality SRH care to them
Accessibility issues	Physical inaccessibility to the health facility by PwDs	Lack of adaptability and compatibility of health infrastructure to meet PWDs’ needs (85.5%) was highlighted as one of the main barriers to the delivery of SRH services to PWDs	“Our consulting rooms are not well structured for patients with disabilities. Like the wheelchairs, we don’t even have walkways for them, maybe walkways for somebody in the wheelchair…, I don’t think we really have them” (Doctor, Kumasi, Female, 32 years).	Government should make it a priority to ensure that health facilities are constructed to be physically accessible to PwDs. Existing ones that are not disability friendly could also be renovated to make them more disability friendly.
Navigating bottlenecks	Compassion and benevolence towards PwDs	Generally, HPs had high scores in the gains domain towards PwDs, meaning they see them as people who can achieve their life goals despite their disabilities. (Gains score = 12.5 ± 3.2). HPs perceived PwDs as people with future prospects. This was demonstrated by the high mean scores of the prospects sub-scale. Prospects score = 15.1 ± 3.0 These could influence HPs’ compassion and benevolence towards them.	“Our work is a calling. So, if you don’t have patience, then you will not be able to ensure the successful implementation of activities to improve SRH services to PwDs” (Midwife, Kumasi, Female, 30 years). “Amidst all of these challenges, we still provide care to PwDs because we perceive that we can be in that situation. Also, at the end of the month, we have to report on our targets. As humans, we always want to win. That is the motivation for us” (Nurse, Offinso North, Female, 37 years).	Healthcare providers adopted compassion and benevolence to circumvent some of the challenges they faced in the delivery of SRH services. Compassion should be a key trait to instill in HPs during their training or while they are schooling to ensure adequate service provision to people, including PwDs. This should be demonstrated during their clinical training/attachment.
	Improvision to keep the job going		“I sometimes write on a piece of paper for those who can read and with those who can’t read, we call some of our staff who have been trained on the use of sign language at their tertiary level to communicate with them. For instance, I have learned sign language so when they come, I use the basics to communicate with them” (Midwife, Kumasi, Female, 56 years). “…I can easily improvise to make room for things that I don’t know. If I have to watch a video on *YouTube* to get conversant with a form of care to PwDs, I can easily do that and work with it. For the visually impaired, they can hear so I have oral conversation with them. But for the deaf and dumb [hearing impaired], you have to use sign language. So, I sometimes watch videos on *YouTube* to communicate with them” (Doctor, Kumasi, Male, 28 years).	HPs should be trained to be innovative to aid in the adoption of different strategies to manage challenging situations. A reward system could also be instituted at various health facilities that recognises HPs who go over and beyond to provide efficient care to motivate other HPs.
	Economic support for PwDs		“Sometimes we plead with the dispensary to serve them the drugs if we see that the person is seriously ill and cannot afford a drug” (Nurse, Kumasi, Female, 59 years). “Yes, sometimes some people living with disabilities are from poor background and some of them might be seriously ill so we sometimes plead on their behalf so that the dispensary can give them the drugs that health insurance doesn’t even cover” (Nurse, Kumasi, Female, 59 years).	NGOs and other philanthropists could support the various hospitals to aid in the SRH services provision to PwDs by contributing to the renewal or economic empowerment of PwDs.
	Educational support, awareness creation and referrals of PwDs		“The situation is compounded for PwDs as most of them are unable to work. So, in such situations, we try to resolve issues of finances by encouraging PwDs to register and enrol onto the national health insurance scheme. We educate them about the importance of registering for the health insurance” (Nurse, Offinso North, Male, 34 years). “For issues that we cannot handle, such as the need for referral or something we channel them to our boss or head of department” (Nurse, Offinso North, Male, 32 years).	Better national awareness should be created about the need to support and include PwDs in SRH health policies through media platforms such as radio and TV and community sensitisation. At the health facility level, HPs should be encouraged to intensify education to PwDs about SRH services and opportunities available. Referrals to the next level of care is also encouraged to avoid further complications. This will aid in the uptake of SRH services and ultimately improve SRH care and achieve impactful SRH outcomes.

## Data Availability

The datasets are available from the corresponding author upon reasonable request.

## Abbreviations

ATDS	Attitude Towards Disability Scale
HPs	health professionals
PwDs	persons with disability
WHO	World Health Organization
SDG	sustainable development goal
